# Recurrent Cholera Outbreaks, Democratic Republic of the Congo, 2008–2017

**DOI:** 10.3201/eid2505.181141

**Published:** 2019-05

**Authors:** Brecht Ingelbeen, David Hendrickx, Berthe Miwanda, Marianne A.B. van der Sande, Mathias Mossoko, Hilde Vochten, Bram Riems, Jean-Paul Nyakio, Veerle Vanlerberghe, Octavie Lunguya, Jan Jacobs, Marleen Boelaert, Benoît Ilunga Kebela, Didier Bompangue, Jean-Jacques Muyembe

**Affiliations:** Santé Publique France, Paris, France (B. Ingelbeen);; European Centre for Disease Prevention and Control, Stockholm, Sweden (B. Ingelbeen, D. Hendrickx);; Institute of Tropical Medicine, Antwerp, Belgium (B. Ingelbeen, M.A.B. van der Sande, V. Vanlerberghe, J. Jacobs, M. Boelaert);; Landesgesundheitsamt Baden-Württemberg, Stuttgart, Germany (D. Hendrickx);; Institut National de Recherche Biomedicale, Kinshasa, Democratic Republic of the Congo (B. Miwanda, O. Lunguya, J.-J. Muyembe);; Utrecht University, Utrecht, the Netherlands (M.A.B. van der Sande);; Ministère de la Santé, Kinshasa (M. Mossoko, B.I. Kebela, D. Bompangue);; Médecins sans Frontières, Kinshasa (H. Vochten, B. Riems, J.-P. Nyakio);; Université de Kinshasa, Kinshasa (D. Bompangue)

**Keywords:** cholera, epidemiology, disease outbreaks, Vibrio cholerae, epidemic history, Democratic Republic of the Congo, bacteria, enteric infections

## Abstract

In 2017, the exacerbation of an ongoing countrywide cholera outbreak in the Democratic Republic of the Congo resulted in >53,000 reported cases and 1,145 deaths. To guide control measures, we analyzed the characteristics of cholera epidemiology in DRC on the basis of surveillance and cholera treatment center data for 2008–2017. The 2017 nationwide outbreak resulted from 3 distinct mechanisms: considerable increases in the number of cases in cholera-endemic areas, so-called hot spots, around the Great Lakes in eastern DRC; recurrent outbreaks progressing downstream along the Congo River; and spread along Congo River branches to areas that had been cholera-free for more than a decade. Case-fatality rates were higher in nonendemic areas and in the early phases of the outbreaks, possibly reflecting low levels of immunity and less appropriate prevention and treatment. Targeted use of oral cholera vaccine, soon after initial cases are diagnosed, could contribute to lower case-fatality rates.

The Democratic Republic of the Congo (DRC) accounts for an estimated 189,000 (5%–14%) of the 1.34–4.01 million cholera cases worldwide annually (*1*,*2*). *Vibrio cholerae* repeatedly reappeared in the DRC throughout the 1970s and became endemic around the Great Lakes in eastern DRC in 1978, resulting in part from favorable conditions for the bacterium’s environmental survival (*3*–*6*). Complex emergencies in eastern DRC have since enabled the regular spread of cholera along the lake banks and to surrounding health zones, driven by water supply interruptions, high population densities, and population movement (*5*,*7*–*9*). In 2017, a countrywide cholera outbreak totaling >53,000 cases and 1,145 deaths was reported in DRC, affecting 20 out of 26 provinces, some of which had not seen cholera cases for more than a decade (*10*).

Cholera prevention and control rely on rapid outbreak detection, access to clean water, safe sanitation, dedicated treatment centers, and the targeted use of oral cholera vaccines (OCV) (*11*). We describe major cholera outbreaks that occurred in DRC during 2008–2017 to explore possible drivers for the spread of cholera in DRC and provide guidance for prevention and control interventions.

## Methods

### Study Design

We performed a retrospective analysis of cholera outbreaks from national cholera surveillance data and reference laboratory data collected from January (week 1) 2008 through November (week 46) 2017. In addition, we analyzed case management data collected during outbreaks in 2015–2017 from a selection of cholera isolation and treatment wards, called cholera treatment centers (CTCs).

### Surveillance Data

Cholera is a notifiable disease in DRC and is therefore included in the national Integrated Disease Surveillance and Response System (IDSRS). The IDSRS is a syndromic surveillance system that compiles weekly morbidity and mortality reports, aggregated at the health zone level. These reports include weekly counts of suspected cholera cases and deaths, stratified into 2 age categories, <5 years and >5 years.

The IDSRS uses 2 case definitions for a suspected cholera case, depending on whether a cholera outbreak has been declared by the Ministry of Health. During an outbreak, the definition is acute watery diarrhea with or without vomiting in a patient >1 year of age; in nonoutbreak situations, the definition is severe dehydration or death following acute watery diarrhea in a patient >5 years of age.

### Other Definitions

**Figure 1:**
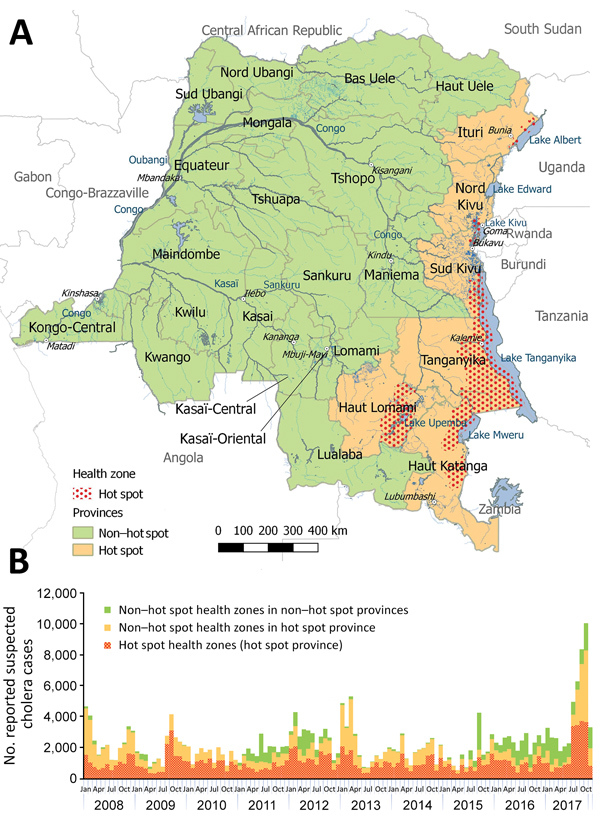
Hot spot and non–hot spot locations for cholera and number of suspected cases by location, Democratic Republic of the Congo, 2008–2017. A) Locations of cholera hot spot and non–hot spot provinces and hot spot health zones (2017 classification). B) Weekly number of suspected cholera cases. Case counts for 2017 are through week 46.

The World Health Organization (WHO) defines cholera hot spots as geographically limited areas where environmental, cultural, or socioeconomic conditions make transmission of disease easier and where cholera persists or reappears regularly (*11*). In DRC, hot spots are defined at the health zone level; 26 (5.0%) of 518 health zones across 6 of 26 DRC provinces are labeled as cholera hot spots according to WHO classification (D. Legros, World Health Organization, pers. comm., 2017 Nov 17). We considered a health zone’s hot spot status to be stable throughout the study period. We defined a hot spot province as a province that included >1 hot spot health zones (Figure 1, panel A). A non–hot spot province was any province that did not contain any hot spot health zones.

We generally defined an outbreak as follows: >1 laboratory-confirmed cholera case and an increase in the number of suspected cases for >3 consecutive weeks. In the 3 provinces that consistently reported cholera cases all year (North Kivu, South Kivu, and Tanganyika), we applied a minimum threshold of 1,000 cases/week for >3 consecutive weeks. In the 2 provinces where sampling for laboratory confirmation was lacking (Ituri and Haut Lomami), we defined a major outbreak as any increase in the number of suspected cases for >3 consecutive weeks, reported by >3 different health zones.

### Microbiological Data

The DRC national cholera reference laboratory, located at the Institut National de Recherche Biomédicale (INRB) in Kinshasa, carried out routine culture confirmation testing for national surveillance and outbreak confirmation purposes during the entire study period. Fecal samples or rectal swabs from patients with suspected cholera, which are usually collected at the beginning or end of suspected cholera outbreaks (*12*), were placed in either Carry-Blair transport medium or on filter paper and transported to the INRB for laboratory confirmation by culture. The following data were extracted from the laboratory database at INRB for each documented clinical sample: age, sex, health zone of residence, date of symptom onset, date of sample collection, date of sample receipt at the reference laboratory, and serotype result. Antimicrobial susceptibility testing was performed, from 2011 onward, by disk-diffusion testing according to Clinical and Laboratory Standards Institute M45-Ed3 (*13*), with testing of erythromycin instead of azithromycin and additional testing of fluoroquinolone antimicrobial drugs. Intermediate-susceptible isolates were grouped with resistant ones.

### Case Management Data

Case management data were provided by 19 CTCs that Médecins sans Frontières had deployed in support of Ministry of Health cholera outbreak response activities, all in non–hot spot health zones, during 2015–2017. Médecins sans Frontières defines a case as >3 liquid stools in the previous 24 hours. From these line lists, we extracted age, sex, health zone of residence, date of symptom onset, date of admission to the CTC, and treatment outcome.

### Population Data

We used population estimates by health zone for 2006 and 2016 provided by the Expanded Programme of Immunization to extrapolate the population of individual health zones for each year during 2008–2017, under the assumption of stable population growth. To ensure comparability of our data throughout the study period, we also used the DRC administrative divisions that were adopted in 2015 (26 provinces, instead of the previous 11) for 2008–2014 data.

### Data Analysis

We analyzed weekly trends in the number of suspected cholera cases reported to the IDSRS, age and sex distributions, and case-fatality rates (CFRs) over the entire period, stratified by cholera hot spot status. We also calculated age and sex distributions for confirmed cases and cases admitted to CTCs, based on the reference laboratory register and CTC data. CFRs for persons with suspected cholera and for admitted patients were calculated with the cholera deaths as numerator (IDSRS data) and the suspected or admitted cholera cases as denominator (CTC data). We described the geographic spread of suspected cholera cases over time by mapping annual cumulative incidence rates by health zone. All reported cumulative incidence rates were expressed as suspected cholera cases per 100,000 population.

We performed data collation, cleaning, and analysis using Microsoft Excel (Microsoft, http://www.microsoft.com), Stata 12.0 (StataCorp LP, https://www.stata.com/stata12), and R (The R Project for Statistical Computing, https://www.r-project.org) software. Maps were generated in QGIS 2.18 (Open Source Geospatial Foundation, https://www.osgeo.org) using OpenStreetMap shapefiles.

### Ethics Statement

We analyzed databases that contained routinely collected and aggregated surveillance data and anonymized laboratory and patient admission data. For the use of patient admission data, we obtained ethics approval (ref. ESP/CE/034/2017) from the Kinshasa University Ethics Committee.

## Results

### General Description of Cholera Cases

During January 1, 2008–November 19, 2017, a total of 270,852 suspected cholera infections and 5,231 cholera-related deaths (CFR 1.9%) were reported in DRC in all 26 provinces. The largest cholera outbreaks were reported in 2008, 2009, late 2011 through 2012, early 2013, and late 2015 through 2017 (Table 1; Figure 1, panel B). Of the 9,510 (3.5%) suspected cholera cases for which the national reference laboratory received samples, 2,941 (30.9%), or 1.1% of all suspected cholera cases reported to the IDSRS, were laboratory confirmed for cholera.

**Table 1:** Suspected cases reported and number of samples collected, tested, and confirmed, countrywide, during cholera outbreaks, Democratic Republic of the Congo, 2008–2017

Location	Period	No. suspected cases				No. samples collected (% positive)				Serotype		
		Age <5 y	Age >5 y	Total		Age <5 y	Age >5 y	Total		Inaba	Ogawa	Hikojima
DRC	Jan 2008–Nov 2017	66,008	204,483	270,852		2,028 (34)	7,482 (30)	9,510 (31)		2,612	274	7
Reported outbreaks* in hot spot provinces												
North Kivu, South Kivu, Tanganyika	Aug–Nov 2009	1,935	9,641	11,652		20 (50)	189 (33)	209 (35)		11	63	0
North Kivu, South Kivu, Tanganyika	Aug–Nov 2017	6,653	14,709	21,362		5 (20)	41 (27)	46 (26)		5	7	0
Haut Katanga	Jan–Mar 2008	1,278	4,712	5,990		3 (67)	16 (50)	19 (53)		7	0	0
Haut Katanga	Jan–Apr 2013	1,935	6,504	8,441		1 (100)	11 (55)	12 (58)		4	3	0
Haut Lomami	Jan–Dec 2014	1,285	3,359	4,644		0	0	0		0	0	0
Ituri	Jan–Sep 2012	828	3,868	4,696		0	0	0		0	0	0
Reported outbreaks* in non–hot spot provinces												
Congo River	Jan 2011–Dec 2012	2,809	11,878	14,686		89 (30)	578 (26)	667 (27)		179	0	0
Congo River	Sep 2015–2017	4,991	20,330	25,422		123 (7)	633 (19)	756 (17)		118	10	0
Kwilu, Kwango, Kasai, Lomami, Sankuru	Jul–Nov 2017	374	2,123	2,497		0	10 (20)	10 (20)		1	1	0

*Defined as >1 laboratory-confirmed cholera cases with evidence of local transmission and an increase in the number of suspected cases for >3 consecutive weeks, or weekly incidence >1,000 cases for >3 consecutive weeks for provinces reporting cases all year round, or increasing number of suspect cases for >3 consecutive weeks if no cholera samples were submitted to the national reference laboratory for testing.

Almost half of all suspected cholera cases (127,642; 47.1%) were reported in the 26 hot spot health zones and 224,212 (82.8%) in hot spot provinces. Of the remaining 46,640 suspected cases that were reported in non–hot spot provinces, 42,340 (90.8%) were reported during the outbreaks in 2011–2012 and 2015–2017.

### Demographic Characteristics of Cholera Case-Patients

In hot spot health zones, 33,477 (26.2%) suspected cholera cases and 589 (28.4%) confirmed cholera cases were in children <5 years of age. In this age group, 23,615 (24.4%) suspected and 44 (14.4%) confirmed cases were reported in non–hot spot health zones in hot spot provinces and 8,916 (19.1%) suspected and 48 (10.8%) confirmed cholera cases in non–hot spot provinces. The median age of patients with confirmed cholera was 10 (interquartile range [IQR] 4–26) years in hot spot health zones, 20 (IQR 8–32) years in non–hot spot health zones in hot spot provinces, and 22 (IQR 10–36) years in non–hot spot provinces. Among CTC admissions in non–hot spot provinces, median age of the patients was 17 (IQR 5–32) years; 23% of those patients were <5 years of age. We observed an increase in the proportion of children <5 years of age admitted to a CTC: 19.0% in the first 4 weeks of the outbreak, >24.7% in weeks 5–8, 27.1% in weeks 9–12, and 34.5% in weeks 13–15. Male patients accounted for 51.4% of confirmed cholera cases and 50.2% of CTC admissions.

### CFRs

The CFR among suspected cholera cases was higher in non–hot spot provinces (4.5%) than in hot spot health zones (1.1%) and non–hot spot health zones located in hot spot provinces (1.8%). The CFR for suspected cases was lower for patients <5 years of age (911/66,008; 1.4%) than for those >5 years of age (4,331/204,483; 2.1%). We observed comparable distributions in CFRs by age for suspected cases when stratified by hot spot status (Table 2). Among CTC admissions in non–hot spot provinces, CFRs increased by age, from 2.4% (43/1,759) among children <5 years of age to 4.3% (32/752) among patients >50 years of age. CFRs decreased throughout an outbreak, from 5.1% (23/452) among CTC admissions in the first week of an outbreak to 4.4% (35/793) in the fifth week and 0.7% (3/429) in the tenth week.

**Table 2:** Case-fatality rate among suspected cholera cases, 2008–2017, and among patients admitted to a cholera treatment center, 2015–2017, Democratic Republic of the Congo

Criterion and location	**Patient age, y**	**No. deaths**	**No. (%) cases**	**Case-fatality rate, %**
Suspected cholera cases				
Overall		5,231	270,852 (100)	1.9
Hot spot health zones	Total	1,407	127,642 (100)	1.1
	<5	292	33,477 (26)	0.9
	>5	1,116	94,082 (74)	1.2
Non–hot spot health zones in hot spot provinces	Total	1,745	96,570 (100)	1.8
	<5	301	23,615 (24)	1.3
	>5	1,440	72,777 (75)	2.0
Non–hot spot provinces	Total	2,079	46,640 (100)	4.5
	<5	318	8,916 (19)	3.6
	>5	1,775	37,624 (81)	4.7
CTC admissions				
Overall		267	9,076 (100)	2.9
Non–hot spot health zones in hotspot provinces	Total	3	1,294 (100)	0.2
	<5	0	357 (28)	0.0
	5–19	1	625 (48)	0.2
	20–49	1	241 (19)	0.4
	>50	1	63 (5)	1.6
Non–hot spot provinces	Total	264	7,782 (100)	3.4
	< 5	43	1,759 (23)	2.4
	5–19	68	2,442 (31)	2.8
	20–49	104	2,609 (34)	4.0
	>50	32	752 (10)	4.3

### Geographic Spread in Hot Spot Provinces

Suspected cholera cases were reported all year in 3 of 6 hot spot provinces: North and South Kivu and Tanganyika, along Kivu and Tanganyika Lakes. Major outbreaks occurred in these provinces in 2009 and 2017. Both outbreaks started in August, peaked 6–8 weeks later, and decreased in intensity until the regions went back to baseline levels 5 months after the peak. Hot spot health zones constituting the lakeside cities of Goma (North Kivu) and Kalémie (Tanganyika) were first to report increasing case numbers, followed by adjacent non–hot spot health zones. The highest annual cumulative incidence among hot spot health zones was reported in Goma in 2017 (1,015 cases/100,000 inhabitants).

In the other 3 hot spot provinces, Ituri, Haut Lomami, and Haut Katanga, more sporadic outbreaks occurred. The largest outbreaks were observed in Haut Katanga in 2008 and 2013. Both outbreaks occurred during January–March; the 2008 outbreak counted 5,990 suspected cases and the 2013 outbreak 7,533 suspected cases. In both instances, most outbreak cases were reported in non–hot spot zones, in the cities of Lubumbashi and Likasi: 5,645 (94%) in 2008 and 6,534 (87%) in 2013. Haut Lomami Province reported smaller, but more frequent, outbreaks (every year, except in 2011); the number of suspected cases varied from 690 in 2009 to 4,644 in 2014. Of the 19,975 suspected cases reported in Haut Lomami Province, 17,043 (85%) were from hot spot health zones around Upemba Lake. In 2017, non–hot spot health zones along the Lualaba River, a tributary of the Congo, also started to report cases. Ituri Province experienced a large outbreak during January–September 2012. Initial cases were reported in hot spot health zones along Lake Albert, followed by marked case increases in neighboring health zones.

### Geographic Spread in Non–Hot Spot Provinces

**Figure 2:**
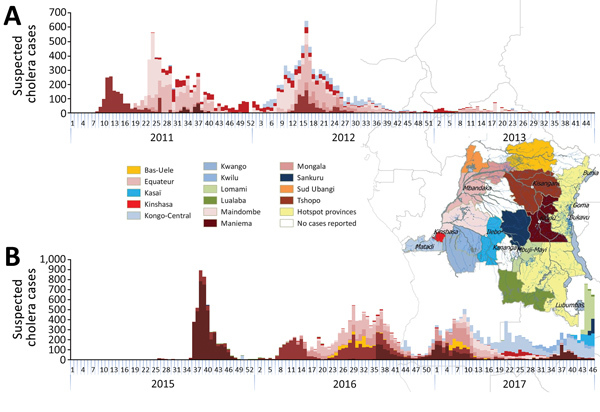
Weekly number of suspected cholera cases for non–hot spot provinces, Democratic Republic of Congo, 2011–2013 (A) and 2015–2017 (B). Colors differentiate provinces and correspond to the colors used in the overlaid map. Case counts for 2017 are through week 46.

In non–hot spot provinces, 2 major recurrent outbreaks occurred along the Congo River, the first in 2011–2012 and the second in 2015–2017. The outbreaks started in 2 different towns located in eastern, upstream provinces through which the Congo River flows: Kisangani (Tshopo) in March 2011 and Kindu (Maniema) in August 2015. From there, both outbreaks gradually progressed downstream in a westerly direction, consecutively reaching health zones in the provinces of Mongala, Equateur, Mai-Ndombe, Kinshasa, and Kongo Central (Figure 2). The elapsed time between the first reported outbreak cases in upstream provinces and those reported in Kongo Central, at the mouth of the Congo River, was 11 months for the 2011–2012 outbreak and 14 months for the 2015–2017 outbreak. Several provinces affected by the 2 outbreaks experienced a second, less intensive peak in suspected cholera cases approximately 1 year after the initial outbreak peaks. This dynamic was observed in several non–hot spot provinces: Tshopo in March 2011 and April 2012, Equateur in June 2011 and April 2012, and Mai-Ndombe in June 2011 and March 2012. Maniema Province experienced 2 such postoutbreak increases following an initial outbreak peak in September 2015: the first in January 2017 and a second in September 2017.

**Figure 3:**
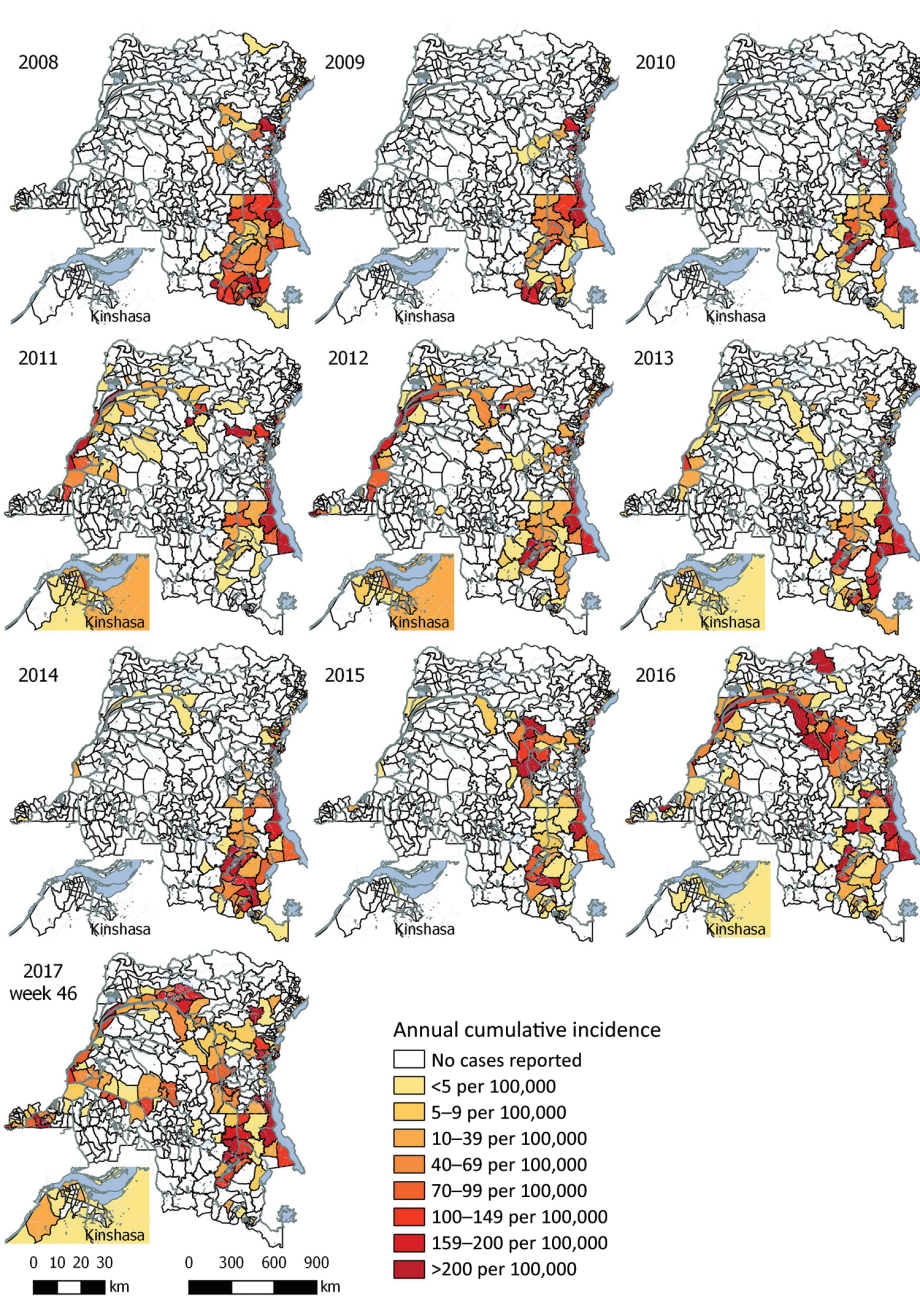
Annual cumulative incidence of suspected cholera cases reported per health zone, Democratic Republic of the Congo, 2008–2017. Case counts for 2017 are through week 46.

In 2017, in addition to the downstream spread along the Congo, suspected cholera cases were reported in inland non–hot spot provinces where no cases had previously been reported during the study timeframe (Figures 2, 3). In July 2017, cases were reported in Kwango Province, followed by Kasaï, Lomami, and Sankuru Provinces, upstream along the Kasaï and Sankuru Rivers.

Along the Congo River, we observed the highest annual cumulative incidence rates in 3 different locations in 3 different years. The first came in 2011 in Bolobo (Mai-Ndombe, 1,107/100,000 population), the second in 2015 in Alunguli (Maniema, 1,088/100,000 population), and the last in 2017 in Kimpese (Kongo Central, 1,335/100,000 population).

### Distribution of Cholera Serotypes

Serotyping data were available for 2,893 (98.4%) laboratory confirmed samples. Overall, Inaba was the most common serotype (90.3%), followed by Ogawa (9.5%) and Hikojima (0.2%) (Table 1). In the 2009 cholera outbreak in North and South Kivu and Tanganyika, the Ogawa serotype was detected in 84.0% of confirmed samples; in the 2017 outbreak, the Ogawa serotype was detected in 58.3% of confirmed samples. In the non–hot spot province outbreaks, the Inaba serotype was detected in 96.4% of confirmed samples. Although Inaba was the dominant serotype in the 2015–2017 nationwide cholera outbreak, Ogawa serotype cases were identified from August 2016 onward, initially in the upstream Maniema Province, followed by reports further downstream in Tshopo Province in October 2016 and Kinshasa in July 2017.

### Antimicrobial Resistance

Antimicrobial resistance testing yielded the following results: 99.2% (2,011 of 2,028 tested) were susceptible to doxycycline, 32.6% (642/1,993) to erythromycin, 99.1% (1,628/1,642) to tetracycline, 0.4% (8/2,029) to cotrimoxazole, and 96.9% (2,009/2,024) to ciprofloxacin. Of the 15 isolates that were resistant to ciprofloxacin, 14 were reported during 2016–2017.

## Discussion

This 10-year retrospective analysis established 3 mechanisms of geographic spread contributing to the acute escalation of cholera in DRC in 2017: strong increases in the number of cases in cholera-endemic areas, the so-called hot spots, around the Great Lakes in eastern DRC; recurrent outbreaks spreading downstream along the Congo River; and the spread along branches of the Congo River that had been cholera free for at least the preceding 10 years. The observed geographic spread supports the hypothesis that the increased numbers of cases in cholera hot spots located along the Great Lakes functioned as incubators for major countrywide outbreaks (*14*–*17*). Coordinated and sustained cholera control interventions in these hot spot areas could be crucial for achieving cholera prevention, control, and elimination in DRC.

The 2011–2012 and 2015–2017 outbreaks followed a similar pattern: a spread from hot spots in the Great Lakes region to major cities located in the upstream section of the Congo River, then progressively spreading downstream, eventually reaching the country’s densely populated capital of Kinshasa and Kongo-Central Province at the mouth of the Congo River. These outbreaks recurred in the same health zones over several years with peaks 1 year apart. Cholera propagation along major rivers has also been observed in Mali, Niger, Sudan, and the Central African Republic (*17*). Population movement and seasonal activities that increase human traffic and trade along the Congo River, and on the Great Lakes in particular, are likely to be key factors in such spread (*16*–*18*).

The range of circulating cholera strains in DRC, their origin, and their contribution to the epidemic remain unclear. During the 2011–2012 outbreak in nonendemic areas along the Congo River, fecal samples collected 1 year apart belonged to a single serotype and multilocus variable number tandem repeat analysis haplotype (*19*), suggesting that this outbreak was caused by a single cholera strain. Samples collected during the first year of the 2015–2017 outbreak affecting the same areas along the Congo River belonged to one serotype. The different serotype isolated 1 year later, which followed the same downstream spread to reach Kinshasa only after a year, probably suggests reintroduction of *V. cholerae*, rather than continued presence of the original *V. cholerae* through asymptomatic human carriers or an environmental reservoir in each of these communities living along the Congo River. In the hot spot provinces of DRC, several *V. cholerae* serotypes were simultaneously identified throughout the study period (Table 1), and isolates from several years grouped in 2 distinct multilocus variable-number tandem-repeat analysis haplotypes (*19*). This finding confirms the presence and role in these provinces of a *V. cholerae* reservoir, either the lakes or potential asymptomatic human carriers (*7*). In addition to gaining further insight into *V. cholerae* circulation, whole-genome sequencing studies could elucidate whether diversification of circulating strains contributed to the intensification of cases in cholera-endemic areas in 2017.

Our findings indicated that cholera outbreaks more disproportionately affected young children, particularly in hot spot provinces where preexisting immunity in the older population was possible. We also found that, although outbreaks along the Congo River affected all ages at the start of the outbreak, the adult population became gradually less affected in the subsequent weeks compared with young children. This finding might suggest that children continued to be exposed more intensely than adults, that fewer adults remained susceptible to become ill after being infected, or that the case definition was less specific for children with watery diarrhea of other origin (non-*Vibrio*).

In a 2017 position paper, WHO recommended the targeted use of OCV in cholera-endemic regions, in humanitarian crises, and during outbreaks (*11*). The use of OCV in DRC has been limited so far to small-scale interventions: 120,000 persons in Kalémie (Tanganyika) in 2015 and 375,000 persons in 5 health zones along the Congo River in Kinshasa in 2016, attaining 90.0% vaccination coverage after a single OCV dose (A. Blake, Epicentre, Paris, pers. comm., 2017 Nov 14). OCV could be considered in several situations in DRC: in cholera hot spot health zones that report a notable increase in reported cases; in non–hot spot health zones adjacent to hot spot health zones, when such increases occur; in health zones along the Congo River, when surveillance reports cholera in upstream communities; during acute emergencies in non–cholera endemic areas of DRC where suspected cases are reported and confirmed; and in settings with particularly poor water and sanitation conditions. In 2015, only 31% of the population in rural DRC used a drinking water source protected from outside contamination, and 29% used sanitation facilities (*20*); targeted OCV can provide an effective additional means to control cholera in areas without good water and sanitation conditions.

Antimicrobial resistance testing results support the continued use of doxycycline to treat severe cholera (*21*) but indicate that cotrimoxazole and erythromycin (and probably azithromycin), which are alternate treatment choices, are unlikely to be very effective treatment options. Ciprofloxacin remains an alternate option to treat children (*22*), but the recent emergence of ciprofloxacin resistance needs to be monitored.

Some limitations apply to our study. Although the reporting of suspected cholera likely does not accurately reflect the full burden of cholera in DRC, it does allow for the documenting of trends and outbreaks. Zero case reporting is not required in the IDSRS, possibly leading to an underestimation of suspected cholera in our analysis, particularly for non–cholera endemic areas where health services might not as readily clinically diagnose and report cholera. Logistical constraints, the lack of an established and systematically implemented national sampling protocol, and limited resources (including an inconsistent availability of transport media) resulted in variable sampling and laboratory confirmation rates over time and place. A more systematic sampling and laboratory confirmation protocol could be developed on the basis of existing international guidelines (*23*) and possibly through the implementation of a decentralized cholera confirmation laboratory network. Our study considered hot spots to be stable throughout 10 years. When observing the affected provinces over the years, we found no indications that hot spots at the province level changed. However, at the health zone level, hot spots could have changed, which could have influenced some of our findings.

Focusing control efforts in DRC on hot spots would be an effective approach to reach elimination only if it can be done rapidly and effectively. Considering the context of conflict and instability in some of the hot spot health zones, a critical portion of the population in the hot spots may continue to be infected, and traveling of cases to nonendemic health zones will then give rise to new outbreaks every few years. Our study was able to identify several such highly vulnerable health zones that are at risk of recurrent outbreaks that could be avoided through the use of OCV, providing population immunity.

In conclusion, 2017 was characterized by an intensified epidemic along the Great Lakes of DRC, recurrence of an outbreak downstream of the Congo River, and an unexpected increase in cholera cases in inland regions of DRC where no cases had been reported for 15 years. Furthermore, ongoing conflicts in the cholera-endemic provinces remain a concern, hampering control efforts at the presumptive origins of outbreaks. Surveillance data adequately describe geographic spread and differences in CFRs, which can be used for targeting of cholera prevention and control actions. A policy for targeted vaccination of at risk populations is needed. Epidemiologic and phylogenetic studies of historical and circulating cholera strains could provide further insight into how cholera spreads from one community to others.
